# Estimating ranibizumab injection numbers and visual acuity at 12 months based on 2-month data on branch retinal vein occlusion treatment

**DOI:** 10.1038/s41598-022-11113-y

**Published:** 2022-05-10

**Authors:** Toshinori Murata, Mineo Kondo, Makoto Inoue, Shintaro Nakao, Rie Osaka, Chieko Shiragami, Kenji Sogawa, Akikazu Mochizuki, Rumiko Shiraga, Takeumi Kaneko, Chikatapu Chandrasekhar, Akitaka Tsujikawa, Motohiro Kamei

**Affiliations:** 1 Department of Ophthalmology, School of Medicine, Shinshu University, 3-1-1 Asahi, Matsumoto, Nagano Japan; 2 Department of Ophthalmology, Mie University Graduate School of Medicine, Mie, Japan; 3 Department of Ophthalmology, Kyorin Eye Center, Kyorin University School of Medicine, Tokyo, Japan; 4 Department of Ophthalmology, Graduate School of Medical Sciences, Kyushu University, Fukuoka, Japan; 5 Department of Ophthalmology, Kagawa University Faculty of Medicine, Kagawa, Japan; 6 Department of Ophthalmology, Asahikawa Medical University, Hokkaido, Japan; 7 Novartis Pharma K.K., Tokyo, Japan; 8 Novartis Healthcare Pvt. Ltd., Hyderabad, India; 9 Department of Ophthalmology and Visual Sciences, Kyoto University Graduate School of Medicine, Kyoto, Japan; 10 Department of Ophthalmology, Aichi Medical University, Aichi, Japan

**Keywords:** Macular degeneration, Retinal diseases

## Abstract

Anti-vascular endothelial growth factor treatment for macular edema secondary to branch retinal vein occlusion generally provides good visual acuity (VA) improvement but may require repeated injections for years. To reduce the number of patients who suffer from avoidable VA loss caused by treatment drop-out, providing prospects of the correlation between expected vision improvement and required number of injections at the early stages of treatment may be helpful. In this post hoc analysis of the phase IV, randomized, open-label ZIPANGU study, we investigated the correlation between the data from Month 2 and Month 12 in terms of VA and required ranibizumab injection numbers. Fifty-nine patients were evaluated (ranibizumab monotherapy, 29; combination therapy, 30). In the monotherapy group, patients who received 1 and 3 injections by Month 2 received a mean total of 2.8 and 8.3 injections during the year, respectively. Data from the combination group were similar. The correlation coefficients for VA scores at Months 2 and 12 were 0.60 and 0.51 for the monotherapy and combination groups, respectively (both p < 0.01). Based on VA and injection numbers at Month 2 of treatment, physicians could provide rough prospects on patients’ expected final VA and required number of injections.

## Introduction

Branch retinal vein occlusion (BRVO) has been reported to be the second most common vision-threatening vascular disorder of the retina after diabetic retinopathy worldwide^1,2^. BRVO threatens vision, largely owing to the development of macular edema (ME)^3,4^ mediated by the breakdown of the blood–retinal barrier by vascular endothelial growth factor (VEGF)^5,6^.

The effects of intravitreal/sub-Tenon triamcinolone injection^7^ or slow-releasing steroid implants^8^ have been widely investigated and these treatments are commonly used in outpatient clinics. A real-world study of 5661 patients reported that patients with macular edema due to BRVO clearly maintained vision longer when treated with anti-VEGF than with either intravitreal steroids or macular laser^9^. Although anti-VEGFs are now established as first-line treatment for ME due to BRVO^10,11^, the need for frequent intraocular anti-VEGF injections may be burdensome for patients both physically and financially^9,12^, and often patients are lost to follow-up for treatment in regular outpatient clinics. In previous studies of anti-VEGF ranibizumab monotherapy, the mean number of injections varied from 8.4 over 12 months (BRAVO study)^13^ and 7 over 12 months (BLOSSOM study)^14^ to 4.8 over 6 months^15^ and 11.4 over 2 years^16^ (BRIGHTER study). In the VIBRANT study of aflibercept, the mean number of monotherapy injections was 9 over 12 months^17^. In addition, intravitreal injection of anti-VEGF agents may be associated with ocular or systemic complications, although the incidence is low^18^. A real-world study based on a Japanese Claims Database (Japan Medical Data Center) reported that the median number of anti-VEGF injections for patients with retinal vein occlusion was 2 per year^19^. Thus, new approaches are warranted to decrease the number of anti-VEGF injections to reduce the burden of patients with BRVO while maintaining treatment efficacy in terms of visual acuity (VA).

The ZIPANGU study was a phase IV, randomized, open-label, active-controlled, 12-month, two-arm, multicenter clinical study designed to investigate whether ranibizumab plus focal/grid laser combination therapy could reduce the number of injections required compared with ranibizumab monotherapy in Japanese treatment-naïve patients with visual impairment due to ME secondary to BRVO^20^.

In the ZIPANGU study, the mean number of ranibizumab injections in the ranibizumab monotherapy arm was successfully reduced to just 4.3 injections over 12 months, while adjunct laser therapy did not result in further reduction of the number of injections (4.1 injections)^20^. These results suggested that the key to a smaller number of ranibizumab injections in the ZIPANGU study may be a shorter disease duration before starting ranibizumab treatment, which was on average 2.2 months (monotherapy group, 2.0 months; combination group, 2.4 months), compared with that in previous clinical trials. For example, in the BRIGHTER study, the average disease duration before treatment was 10.3 months in the monotherapy group and 9.2 months in the combination group^15,16^. In the RABAMES study, the average disease duration was 5 months in the grid laser group, 5.1 months in the ranibizumab monotherapy group, and 6.0 months in the combination group^21^. In the RELATE study, it was 12.7 months in the ranibizumab monotherapy group^22^.

The results from ZIPANGU confirmed that a 1 + *pro re nata* (PRN) ranibizumab monotherapy regimen in the first year for patients with ME secondary to BRVO led to good VA outcomes with a smaller number of ranibizumab injections than that reported in previous studies, provided that the patients had a short duration of vision deterioration at an average of 2 months. Under these conditions, the patients achieved vision improvement by 22 Early Treatment in Diabetic Retinopathy Study (ETDRS) letters. A report by the American Academy of Ophthalmology suggested that central subfield foveal thickness (CSFT) reduction was similar even with delayed anti-VEGF treatment in BRVO. However, the functional response did not rebound to the same level as eyes treated early using anti-VEGF agents^23^. Conversely, adjunctive focal/grid laser did not significantly reduce the number of ranibizumab injections in combination-treated patients compared with those receiving monotherapy^20^. Based on the ZIPANGU study findings, information on disease duration may be the key to identifying patients who would most benefit from ranibizumab monotherapy.

However, early treatment for BRVO patients with ME is often difficult because BRVO patients tend to wait for a relatively long time before seeing a physician^15^. Usually, BRVO patients do not consider their vision deterioration to be severe, partly because they maintain good vision in the non-affected eye. They usually decide to wait and see if there is spontaneous vision recovery. This may be the reason, at least in part, why the patients included in previous studies had a longer disease duration than those in the ZIPANGU study (average of 2 months) as described above^15,20–22^.

For this reason, we performed a post hoc analysis of the ZIPANGU study to assess which factors other than the duration of the disease could help identify which BRVO patients may be able to achieve good final vision recovery with an average of 4 ranibizumab injections per year. We performed this post hoc analysis focusing on CSFT, VA, and the number of ranibizumab injections in the early stages of treatment. First, we investigated the correlation between baseline VA and final VA. Then, we chose Month 2 as the time point for the early evaluation of these factors because this was when the average CSFT was reduced to the upper limit of the normal range (i.e., ≤ 300 µm) in most patients. Finally, we investigated the correlation between the data at Month 2 and Month 12 regarding VA and the required number of ranibizumab injections.

## Results

### Patients

In ZIPANGU, 59 patients were randomly assigned to treatment (ranibizumab monotherapy, n = 29; combination therapy, n = 30). The mean age ± standard deviation (SD) was 66.8 ± 9.8 years, the VA was 53.5 ± 11.6 letters, the CSFT was 558.2 ± 164.6 μm, and the mean duration since onset of BRVO was 2.2 ± 1.2 months. Values were similar between treatment groups.

Overall, 44 patients (23 in the monotherapy arm and 21 in the combination arm) had onset of BRVO within < 3 months prior to the start of treatment, 15 patients (six and nine, respectively) had onset within ≥ 3 to < 6 months, and no patients had BRVO onset ≥ 6 months prior to the start of treatment. At baseline, a total of 36 patients (17 in the monotherapy arm and 19 in the combination arm) had macular ischemia within the 6-mm perifoveal subfield, 41 (17 and 24, respectively) presented subretinal fluid, and 57 (29 and 28, respectively) presented intraretinal fluid.

### Impact of the number of injections up to 2 months on the total number of injections up to 1 year

**Figure 1:**
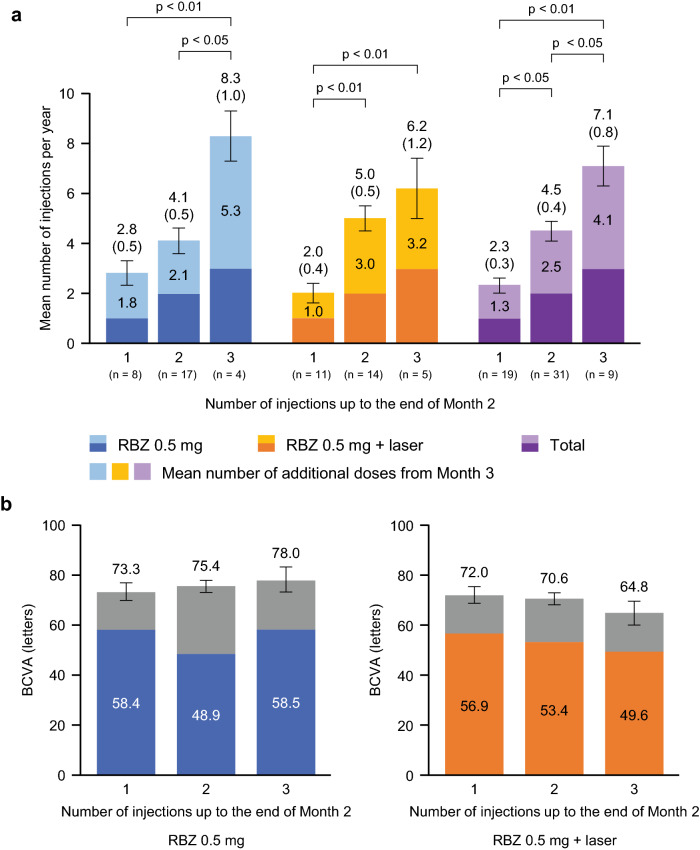
(**a**) Number of ranibizumab injections per year (SE) according to the number of injections administered up to the end of Month 2 (safety analysis set). (**b**) Corresponding mean (SE) visual acuity (letters) at Month 12. p values obtained from *t*-test with Tukey’s method. By analysis of variance, for 1 vs 2 vs 3 injections, p < 0.01. In (**b**), the colored bars represent the BCVA at baseline and the gray bar represents the BCVA at Month 12. *BCVA* best corrected visual acuity, *RBZ* ranibizumab, *SE* standard error.

The mean number of ranibizumab injections administered per year according to the number of injections administered up to the end of Month 2 is shown in Fig. 1a. Among all 59 patients with ME secondary to BRVO, patients who received only 1 injection (n = 19) between baseline and Month 2 required a mean ± SD of 2.3 ± 1.4 injections during the first year of treatment. In contrast, patients who received 3 injections (n = 9) during the first 2 months went on to receive a mean ± SD of 7.1 ± 2.4 injections during the first year of treatment (p < 0.01). The group that received 2 injections between baseline and Month 2 received a mean ± SD of 4.5 ± 2.0 injections during the first year of treatment, which was a higher number of injections compared with that of the group that received only 1 injection between baseline and Month 2 (p < 0.05), and a lower number of injections compared with that of the group that received 3 injections between baseline and Month 2 (p < 0.05).

We also analyzed the correlation between the number of ranibizumab injections administered per year and the number of injections administered up to the end of Month 2 by dividing patients into monotherapy and combination therapy groups. In the monotherapy group, patients who received a single injection between baseline and Month 2 were found to receive a mean ± SD of 2.8 ± 1.5 injections in 1 year. In contrast, patients who received 3 injections during the first 2 months went on to receive a mean ± SD of 8.3 ± 1.9 injections in 1 year (p < 0.01). The patients who received 2 injections between baseline and Month 2 received a mean ± SD of 4.1 ± 2.1 injections in 1 year, which was a higher number of injections than that of the group that received 1 injection between baseline and Month 2 (statistically not significant), but a lower number of injections than that of the group that received 3 injections between baseline and Month 2 (p < 0.05). Data from the combination group were similar, with a single early injection associated with a mean ± SD of 2.0 ± 1.3 annual injections and 3 early injections associated with a mean ± SD of 6.2 ± 2.6 annual injections. Of note, VA at Month 12 remained similar regardless of the number of injections received up to the end of Month 2 (Fig. 1b). In addition, among all 59 patients treated, 44% did not receive ranibizumab injections during the latter 6 months of the first year of anti-VEGF treatment.

### The time course of CSFT changes from baseline to Month 12

**Figure 2:**
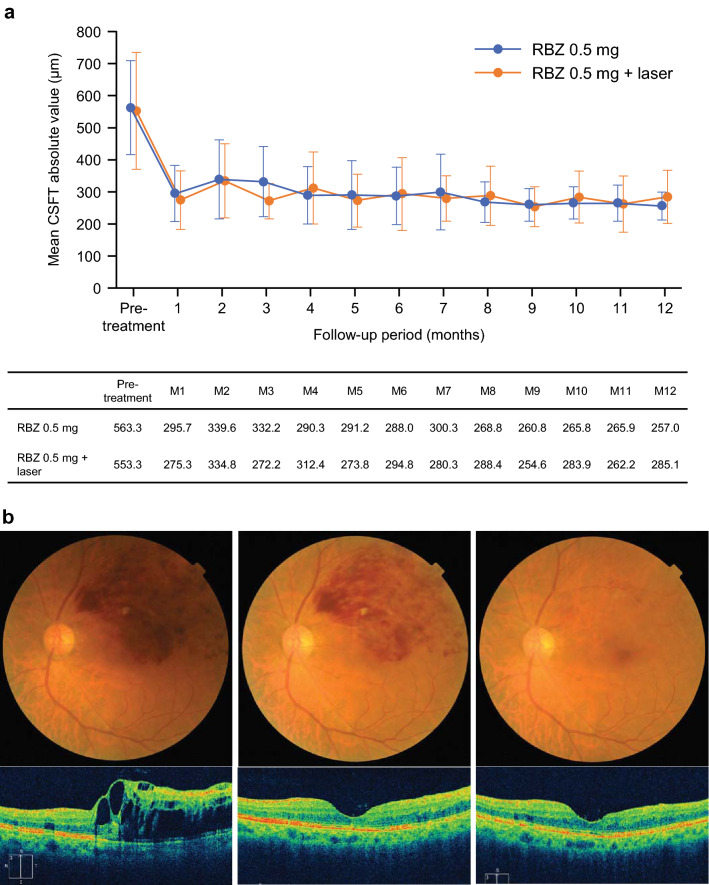
(**a**) Changes in central subfield foveal thickness (CSFT) absolute values over time in all patients. (**b**) A representative case that showed drastic reduction of CSFT and visual acuity (VA) improvement at Month 1, which were maintained up to Month 12 by 1 + PRN ranibizumab injection. In Fig. 1b: Left panel: Baseline fundus photograph and optical coherence tomography (OCT). Hemorrhage is present in the upper half of the macula. OCT shows prominent macular edema (ME). CSFT was 642 μm. VA was 20/50. Middle panel: Month 1. Macular hemorrhage is still present, but OCT shows marked reduction of ME. CSFT was 228 μm. VA was 20/28. Right panel: Month 12. Macular hemorrhage completely resolved. CSFT was 234 μm and OCT showed a well-preserved ellipsoid zone in the fovea. VA was 20/17 (88 ETDRS letters). *RBZ* ranibizumab.

The average baseline CSFT was 563.3 (302–929) μm in the monotherapy group and 553.3 (304–1013) μm in the combination therapy group (Fig. 2a). After a single ranibizumab injection, the average CSFT improved to less than 300 μm, with 295.7 (164–596) μm in the monotherapy group and 275.3 (179–657) μm in the combination therapy group at Month 1.

Fundus photographs and optical coherence tomography (OCT) images of a representative case are shown in Fig. 2b. At baseline, hemorrhage was present in the upper half of the macula, and OCT showed prominent ME with a CSFT of 642 μm. VA was 20/50 (65 ETDRS letters) (left panel). One month after the ranibizumab injection, macular hemorrhage was still present, but OCT showed a marked reduction of CSFT to 228 μm, which is less than the reinjection criteria of 300 μm for ranibizumab. VA improved from 20/50 to 20/28 at Month 1 (middle panel). After 1 + PRN ranibizumab regimen for 1 year, VA improved to 20/17, and hemorrhage in the macula was completely resolved at Month 12. CSFT was 234 μm, and OCT showed a well-preserved ellipsoid zone in the fovea (right panel) with a VA of 20/17 (88 ETDRS letters).

### Correlation between VA at Month 12 and baseline VA or VA at Month 2

**Figure 3:**
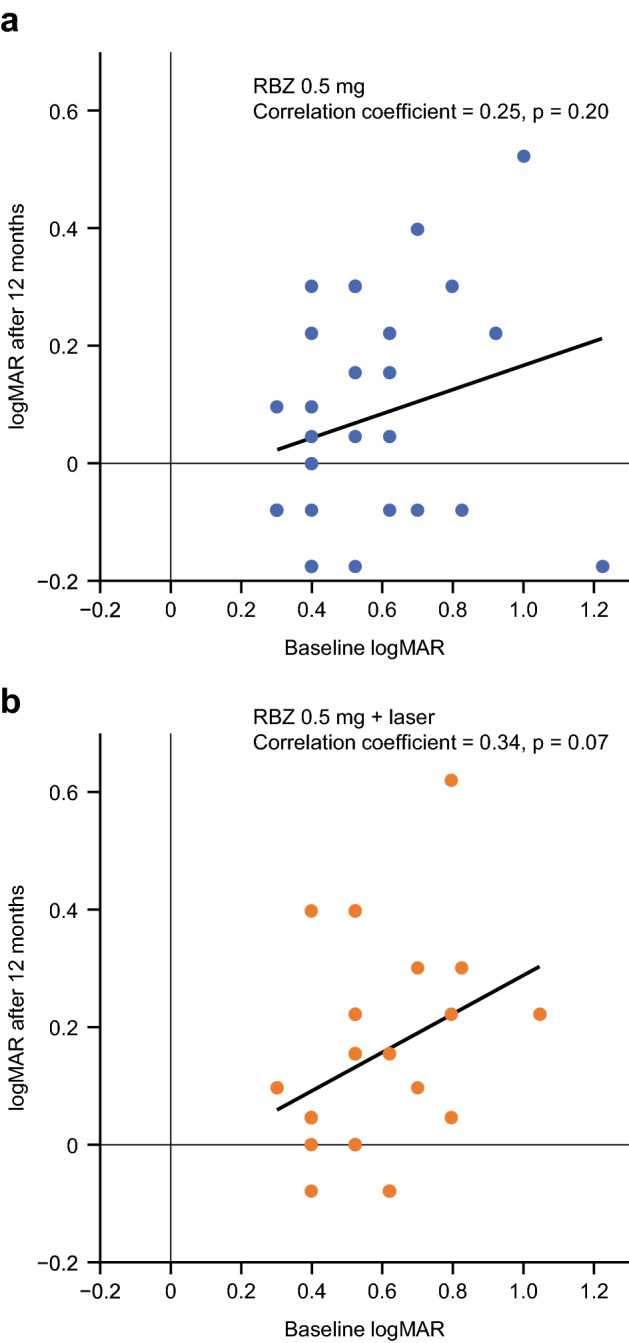
Correlation between visual acuity scores before the start of treatment and at Month 12. (**a**) Ranibizumab 0.5 mg monotherapy. (**b**) Ranibizumab 0.5 mg plus laser. *logMAR* logarithm of the minimum angle of resolution, *RBZ* ranibizumab.

**Figure 4:**
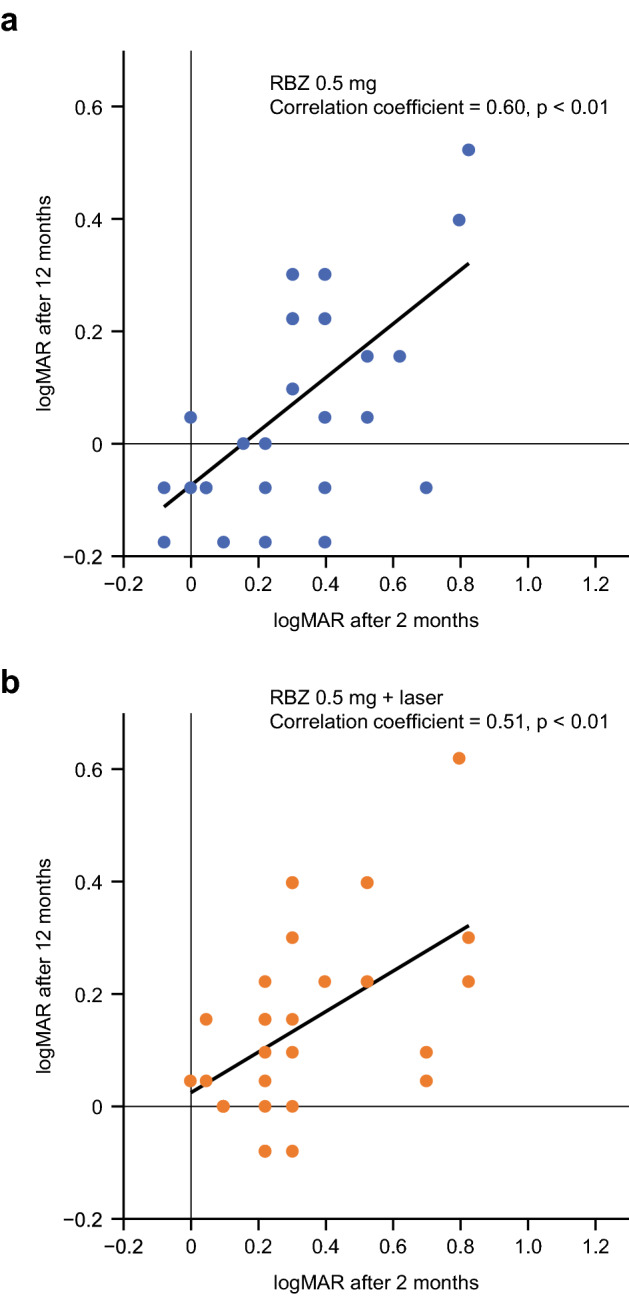
Correlation between visual acuity scores at the end of Month 2 and at Month 12. (**a**) Ranibizumab 0.5 mg monotherapy. (**b**) Ranibizumab 0.5 mg plus laser. *logMAR* logarithm of the minimum angle of resolution, *RBZ* ranibizumab.

VA at baseline did not correlate with VA at Month 12 (Table 1, Fig. 3); the correlation coefficient was 0.25 (p = 0.20) in the monotherapy group and 0.34 (p = 0.07) in the combination therapy group. However, there was a significant correlation between VA at the end of Month 2 and VA at Month 12. The correlation coefficient was 0.60 (p < 0.01) in the monotherapy group and 0.51 (p < 0.01) in the combination therapy group (Table 1, Fig. 4). From Month 2, the average CSFT of most patients decreased to < 300 μm, except for Month 12. Of note, at Month 1 in all three groups, all patients had undergone the baseline ranibizumab injection 1 month prior.

**Table 1:** BCVA logMAR correlations between baseline or Month 2 and Month 12.

Time vs Month 12	Correlation coefficient (p value)		
	Ranibizumab 0.5 mg	Ranibizumab 0.5 mg plus laser	All patients
Baseline	0.25 (0.20)	0.34 (0.07)	0.28 (0.03)
Month 2	0.60 (< 0.01)	0.51 (< 0.01)	0.56 (< 0.01)

*BCVA* best corrected visual acuity, *logMAR* logarithm of the minimum angle of resolution.

### Impact of the number of injections up to 2 months on the final VA

**Figure 5:**
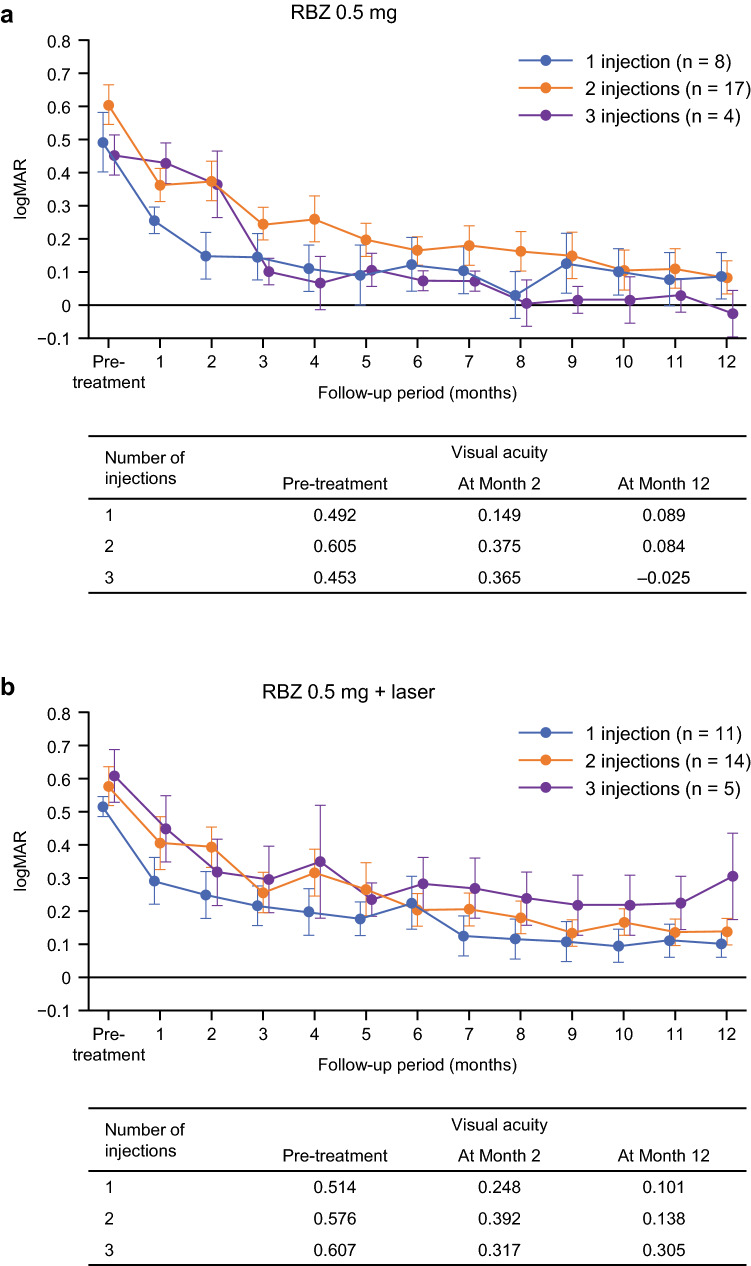

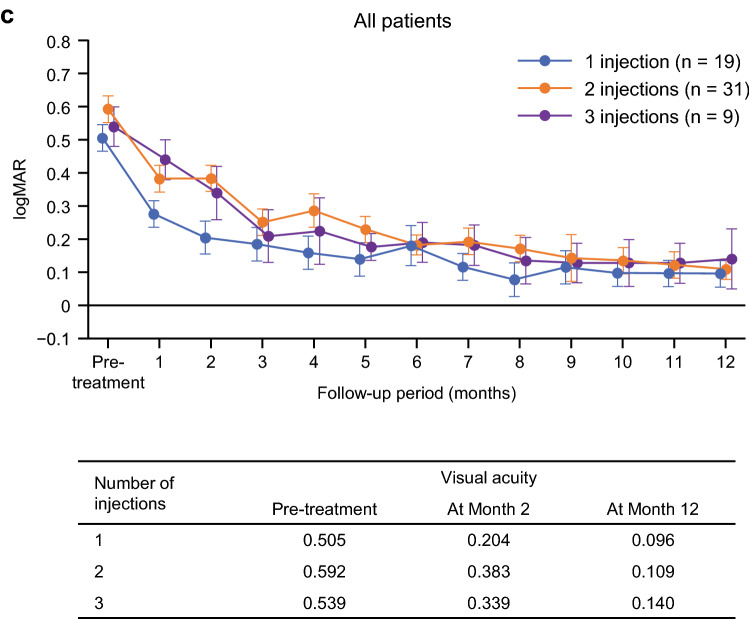
Mean (SE) changes in visual acuity (logMAR) in relation to the number of injections up to 2 months after the start of treatment. (**a**) Ranibizumab 0.5 mg monotherapy. (**b**) Ranibizumab 0.5 mg plus laser. (**c**) All patients. *logMAR* logarithm of the minimum angle of resolution, *RBZ* ranibizumab, *SE* standard error.

There was no significant difference in the time course of VA improvement and final VA at 12 months (monotherapy, p = 0.58; combination, p = 0.11; total, p = 0.85) in relation to the number of injections (1, 2, or 3) up to 2 months (Fig. 5).

**Figure 6:**
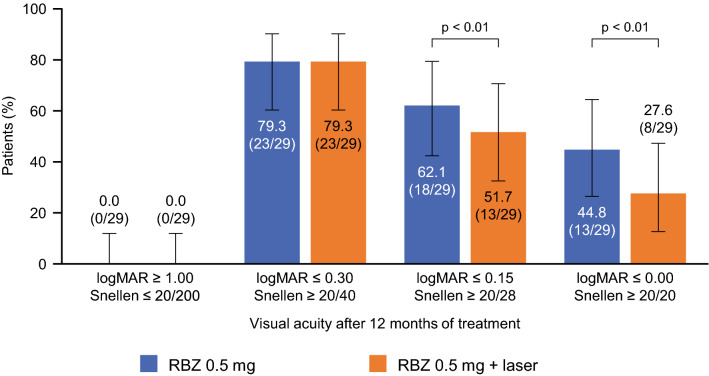
Overall visual acuity performance by treatment group analyzed by logMAR conversion. Number of injections per year: p < 0.01; 1 injection vs 3; *t*-test by Tukey’s method. Error bars represent 95% confidence intervals. *logMAR* logarithm of the minimum angle of resolution, *RBZ* ranibizumab, *Snellen* Snellen visual acuity.

The percentage of patients who achieved logarithm of the minimum angle of resolution (logMAR) VA of ≤ 0.15 and ≤ 0.00 after 12 months was significantly higher in the ranibizumab 0.5 mg monotherapy group compared with the laser combination group (p < 0.01) (Fig. 6). Eighteen of the 29 patients in the monotherapy group (62.1%) achieved decimal VA of ≥ 0.7 (20/29), which is required to obtain and renew a driver’s license in Japan. Notably, 44.8% of the patients in the ranibizumab 0.5 mg monotherapy group achieved a logMAR score of ≤ 0.00 (equivalent to Snellen VA ≥ 20/20) (Fig. 6). Furthermore, in both groups, the percentage of patients with a logMAR of ≥ 1.00 (equivalent to Snellen VA of ≤ 20/200) was 0%.

The number of injections per year and logMAR values after 2 months and 12 months in relation to the number of injections up to 2 months are shown in Table 2.

**Table 2:** The number of injections per year and logMAR values after 2 months and 12 months in relation to the number of injections up to 2 months.

First 2 months	Number of injections per year (average value)	Snellen vision logMAR conversion at 2 months (average value)	Snellen vision logMAR conversion at 12 months (average value)
1 injection	2.3	20/32 logMAR 0.204	20/25 logMAR 0.096
2 injections	4.5	20/48 logMAR 0.383	20/26 logMAR 0.109
3 injections	7.1	20/44 logMAR 0.339	20/28 logMAR 0.140

*logMAR* logarithm of the minimum angle of resolution.

### Impact of the number of injections up to Month 2 on the time course of CSFT changes

In the monotherapy group, the mean ± SD CSFT was 530.4 ± 155.0, 601.1 ± 144.2, and 468.3 ± 106.9 µm at baseline and 236.5 ± 43.5, 259.9 ± 44.4, and 291.5 ± 22.0 µm at Month 12 in patients who received 1, 2, and 3 injections up to Month 2, respectively (Supplementary Fig. S1). In the combination group, the mean ± SD CSFT was 474.9 ± 144.3, 544.8 ± 157.1, and 749.6 ± 208.7 µm at baseline (p = 0.01 for 1 vs 2 vs 3 injections up to Month 2) and 254.5 ± 63.6, 281.1 ± 68.0, and 350.3 ± 140.0 µm at Month 12 in patients who received 1, 2, and 3 injections up to Month 2, respectively. In the total group, the mean ± SD CSFT was 498.3 ± 147.4, 575.7 ± 150.3, and 624.6 ± 219.2 µm at baseline and 246.5 ± 54.8, 269.8 ± 56.6, and 320.9 ± 97.9 µm at Month 12 (p = 0.03 for 1 vs 2 vs 3 injections up to Month 2) in patients who received 1, 2, and 3 injections, respectively. In the monotherapy, combination therapy, and total groups, the CSFT value was always lowest in the group that required 1 injection in the first 2 months to maintain CSFT 300 µm, followed by the group that received 2 injections. The group with the highest CSFT value was that which required 3 injections. By the first 2 months of treatment, the absolute CSFT reached an average of around 300 µm and was maintained for 1 year in all groups by 1 + PRN ranibizumab injections.

## Discussion

In the ZIPANGU study, ranibizumab monotherapy for treatment-naïve patients with ME secondary to BRVO led to 22 ETDRS letter improvement with 4.3 ranibizumab injections, which indicates better VA gains with a lower number of ranibizumab injections than that in previous randomized controlled trials such as the BRAVO study (18.3 letters with 8.4 ranibizumab injections/year) or the BRIGHTER study (15.5 letters with 11.4 ranibizumab injections/2 years)^13,15,16^. The purpose of this post hoc analysis was to identify the factors that led to such a favorable clinical course of treatment in the ZIPANGU study, which could be identified at early stages of the treatment.

Multivariate analyses that assessed the annual number of injections according to the presence of various baseline factors, including baseline VA, retinal ischemia, and OCT findings, failed to identify markers associated with a favorable clinical course of treatment (data not shown). In contrast, previous reports indicated that the baseline VA was correlated with the final VA after ranibizumab treatment for ME secondary to BRVO^24^. Generally, a good baseline VA tends to be associated with a good final VA. However, in this post hoc analysis of the ZIPANGU study, we confirmed that there was no correlation between VA at baseline and VA at Month 12 (Fig. 3). To determine the reason for this finding, we assessed the differences in longitudinal CSFT changes between the ZIPANGU study and previous studies using ranibizumab monotherapy for ME secondary to BRVO. The baseline CSFT varied widely. The average baseline CSFT was 563.3 (302–929) μm in the monotherapy group and 553.3 (304–1013) μm in the combination therapy group. The mean CSFT decreased to the normal range at Month 1 after ranibizumab injection and was maintained at 300 μm or less by additional ranibizumab injection. This rapid normalization of CSFT after a single ranibizumab injection appeared to be the key to a rapid VA improvement in the ZIPANGU study, thus avoiding photoreceptor damage due to persistent ME (Fig. 5).

A lack of correlation between baseline VA and final VA after treatment or rapid decrease of CSFT after a single ranibizumab injection was observed in patients with BRVO in the ZIPANGU study, and these patients had a short disease duration of 2 months on average. If ME persists for a more extended period before and after the initial ranibizumab injection, photoreceptor damage becomes more severe, and good vision recovery may not be expected. Long-lasting ME may damage photoreceptors^23^. In patients with BRVO with a longer disease duration before the initiation of anti-VEGF treatment, CSFT normalization took about 3–4 months after ranibizumab injections in previous studies^15,16,22^. It has been reported that the longer duration of macular edema due to BRVO leads to the formation of an increased number of leaking microaneurysms in the fovea which results in refractory foveal edema and a need for more anti-VEGF injections to reduce CSFT to normal levels^25,26^. The disease duration before starting ranibizumab injections in the monotherapy group was 10.3 months in the BRIGHTER study^15,16^, 12.7 months in the RELATE study^22^, and 3.5 months in the BRAVO study (17.5% had a disease duration longer than 6 months)^13^. Thus, a short disease duration together with a rapid decrease in CSFT after a single ranibizumab injection appeared to be the reason why better vision recovery was achieved in the ZIPANGU study compared with previous studies. These data indicated that poor baseline VA should not be a reason to hinder receiving ranibizumab treatment in eyes with BRVO if the disease duration is short, i.e., around 2 months.

We investigated the correlation between CSFT, VA, and the number of ranibizumab injections at Month 2 and Month 12. We chose Month 2 as the timing for early data analysis based on the results on CSFT changes. At baseline, the average baseline CSFT exceeded 300 μm in all cases, leading to VA deterioration. As described above, the average CSFT was 563.3 (302–929) μm in the monotherapy group and 553.3 (304–1013) μm in the combination therapy group. After the first dose of ranibizumab was administered at baseline, the average CSFT rapidly improved to less than the reinjection criteria of 300 μm and was 295.7 (164–596) µm in the monotherapy group and 275.3 (179–657) µm in the combination group at Month 1. This rapid reduction in CSFT may lead to a good VA improvement regardless of the poor baseline vision due to ME. However, the data at Month 1 in this ZIPANGU study are not suitable for analysis because all the patients underwent ranibizumab injections in the previous months, which is rare during the 1 + PRN regimen in an outpatient clinic setting. In addition, some patients required the second ranibizumab injection at Month 1 to achieve this level of CSFT reduction. Thus Month 2 was selected for data analysis for comparison with the final data at Month 12.

Final VA at Month 12 was good regardless of the number of injections received up to the end of Month 2 (Fig. 1b), suggesting that as long as the CSFT is kept within the normal range with ranibizumab (1 + PRN regimen), a good average VA outcome can be expected. While VA outcomes differ in each patient, there was a correlation between VA at Month 2 and Month 12 both in the monotherapy group (correlation coefficient = 0.60, p < 0.01) and combination therapy group (correlation coefficient = 0.51, p < 0.01) (Fig. 4). These data suggest that patients can be provided a rough prospect of their final VA based on their VA at Month 2.

We divided patients from both treatment arms into three groups depending on the number of injections in the early phase, that is, 1, 2, or 3 injections between baseline and Month 2. Because the final VA was similar among the three groups, we then investigated which group could obtain a good VA recovery with a minimal number of ranibizumab injections. Among all 59 patients, those who received just 1 injection between baseline and Month 2 required only 2.3 injections up to Month 12. In contrast, patients who required 3 injections during the first 2 months went on to receive a mean total of 7.1 injections up to Month 12. In addition, the group that received 2 injections between baseline and Month 2 received a mean total of 4.5 injections up to Month 12, which was a higher number of injections compared with that of the group that received only 1 injection between baseline and Month 2, and a lower number of injections compared with that of the group that received 3 injections between baseline and Month 2.

Based on these post hoc data from the ZIPANGU study, physicians may be able to provide to patients a rough estimation of their expected VA outcomes and the likely number of ranibizumab injections they will require in the first year, as early as 2 months after the start of the treatment. Furthermore, among all patients in this post hoc analysis, 44% of patients did not require additional ranibizumab injections in the latter 6 months of the first year (data not shown), suggesting that these patients may not require further ranibizumab injections in the second year of treatment.

Patients tend to drop out from treatment with anti-VEGF when it is uncertain how long the anti-VEGF therapy will continue and how much their vision will improve. This issue could be ameliorated by informing patients of their expected final vision improvement and the estimated number of ranibizumab injections as early as Month 2 based on these post hoc analysis data.

The present study has some limitations, including the open-label design of the primary study, post hoc methodology for these analyses, and small patient numbers, which made further sub-analysis difficult (e.g., by hypertension and age). Additionally, direct comparisons could not be made between the efficacies of the 1 + PRN regimen with ranibizumab and other ranibizumab dosing strategies such as the treat-and-extend regimen. Finally, as patients in the present study were required to attend study visits every month, it is unknown whether this is also possible in real-world clinical practice.

In conclusion, for patients with ME secondary to BRVO, a poor baseline VA should not be the reason to hesitate to start anti-VEGF therapy because they could expect good VA recovery provided the disease duration is short, and photoreceptors are not damaged severely. After starting anti-VEGF therapy, it may be possible to provide patients with a rough estimate of the expected number of injections needed in the first year and final VA, based on the number of ranibizumab injections administered up to Month 2 and VA at Month 2, respectively. Such estimations may help in managing patients’ motivation to continue treatment and prevent interruptions or drop-outs from treatment. Thus, by providing this information to patients, we may be able to achieve better VA outcomes in patients with ME secondary to BRVO.

## Methods

### Study design and patients

Full details of the ZIPANGU study (NCT02953938) have been described^20^. In brief, 59 Japanese patients with ME secondary to BRVO were enrolled and randomly assigned (1:1 ratio) to receive ranibizumab monotherapy or ranibizumab plus focal/grid short-pulse laser combination therapy. Randomization was balanced by VA (< 0.3 or ≥ 0.3) as assessed by decimal VA/58 letters by the ETDRS chart^27^.

The key inclusion criteria were visual impairment due to ME secondary to BRVO; best corrected visual acuity (BCVA) 0.05–0.5 (ETDRS: 19–73 letters/logMAR: 1.30–0.3); CSFT > 300 μm; and disease duration ≤ 6 months. The key exclusion criteria were a history of treatment with anti-VEGF agents prior to baseline; a history of direct/grid laser to the study eye prior to baseline; and a history of stroke or myocardial infarction within 3 months before screening. Patients with macular BRVO were excluded from this study.

The study was conducted in compliance with the Declaration of Helsinki and Good Clinical Practice guidelines, as well as all applicable local and national regulatory requirements. Approval for the study protocol and all associated documentation was obtained from the Institutional Review Board/Independent Ethics Committee at each study center. Patients provided written informed consent prior to inclusion in the study.

### Treatment

Ranibizumab was administered as a 1 + PRN regimen, which comprised a single 0.5-mg loading dose plus additional monthly doses as needed, with a minimum gap of 30 ± 7 days between injections. Laser treatment was administered at least 30 min before the ranibizumab injection or could be deferred for up to 14 days after the injection, focused within vascular arcades. Further laser treatment was administered at minimal intervals of 30 ± 7 days.

The details of the laser methods were previously reported^20^. Briefly, the focal/grid laser method was a modified version of the ETDRS procedure that incorporates a short-pulse laser to minimize retinal tissue damage, thus avoiding atrophic creep. Focal/direct short-pulse laser treatment was performed on individual microaneurysms, clusters of microaneurysms with fluid retention or leakage, and other leakage points such as enlarged capillaries. Grid short-pulse laser treatment was performed to suppress excess production of VEGF by alleviating retinal ischemia. To achieve this, the entire capillary non-perfusion area in the vascular arcade was covered with grid short-pulse laser treatment. OCT and fluorescein angiography were used to depict the capillary non-perfusion area.

### Study outcomes

The primary study endpoints have been described^20^. For this post hoc analysis, outcomes of interest were: (1) the number of ranibizumab injections per year according to the number of injections administered up to the end of Month 2 and the patterns of injections over time; and (2) the correlation between VA at Month 2 and Month 12. These outcomes were evaluated using decimal VA and the corresponding logMAR.

We undertook an analysis of trends in the overall improvement in VA by evaluating the proportion of patients who achieved good VA, according to a decimal VA of 0.5, 0.7, and 1.0 (which corresponds to logMAR 0.30, logMAR 0.15, and logMAR 0.00, respectively), or poor VA (decimal VA of ≤ 0.1 [legal blindness]; which corresponds to logMAR 1.00).

Ocular adverse events (AEs) consisted largely of ocular surface changes; transient ‘intraocular pressure increased’ was the only one that occurred in > 1 patient in either arm (n = 3, 10.0%). No serious ocular AEs or deaths were reported. The details of the safety outcomes have been reported previously^20^.

### Statistical methods

The power calculations for the main study have been described^20^. As this was a post hoc analysis, and no hypothesis testing was performed, all statistical analyses were conducted at the nominal level. The full analysis set included all randomized patients who received at least one ranibizumab injection. The safety set included all patients who received at least one ranibizumab injection and had at least one post-baseline safety assessment.

Changes in BCVA and CSFT from baseline to Month 12 in patients according to BCVA and VA improvements at Month 12 were evaluated for the following groups: BCVA ≥ 73, ≥ 80, and ≥ 85, and VA improvement ≥ 10, ≥ 15, and ≥ 30 resulting in nine subgroups (i.e., BCVA ≥ 73 and VA improvement ≥ 10, BCVA ≥ 73 and VA improvement ≥ 15, BCVA ≥ 73 and VA improvement ≥ 30, etc.).

Summary statistics are reported for continuous variables (n, mean, median, SD, standard error, Q1, Q3, and min and max values) and the number and percentage of patients for categorical or binary variables. Pairwise comparisons between treatment groups were conducted using a *t*-test with Tukey’s method. Analysis of variance was used to compare weighted averages of the number of injections among injection pattern subgroups within each treatment group. Chi-square or Fisher’s exact test was used to generate statistical comparisons for combination therapy vs monotherapy within each subgroup evaluated. Two-sided 95% confidence intervals were based on exact methods for unadjusted individual percentages and normal approximation for differences in percentages. The Cochran–Mantel–Haenszel test was used for general association statistics. Stratification for the main analysis was based on baseline logMAR BCVA (< 0.52, ≥ 0.52) for analysis of covariance. The Pearson correlation (r) and p values under the null hypothesis of zero correlation are presented for the endpoints in each treatment group. In this analysis, r represents the correlation value obtained using the Pearson correlation method, and the p values were obtained using Fisher's exact method. All tests were two-sided. All analyses were performed using SAS version 9.4 (SAS Institute Inc., Cary, NC, USA).

## Supplementary Information

Supplementary Figure S1.

## Data Availability

All relevant data generated or analyzed during this study are included in this published article and its supplementary materials.
