# Supplementary material for: Estimating ranibizumab injection numbers and visual acuity at 12 months based on 2-month data on branch retinal vein occlusion treatment
Source: Sci Rep. 2022 May 10;12:7661. doi: 10.1038/s41598-022-11113-y (PMC9090796; doi:10.1038/s41598-022-11113-y)
Supplement: Supplementary file 1 — Supplementary Figure S1. [file 41598_2022_11113_MOESM1_ESM.pdf]

Supplementary Materials

Estimating ranibizumab injection numbers and visual acuity at 12 months based on 2-month data on branch retinal vein occlusion treatment

Toshinori Murata, Mineo Kondo, Makoto Inoue, Shintaro Nakao, Rie Osaka, Chieko Shiragami, Kenji Sogawa, Akikazu Mochizuki, Rumiko Shiraga, Takeumi Kaneko, Chikatapu Chandrasekhar, Akitaka Tsujikawa, Motohiro Kamei

Supplementary figures

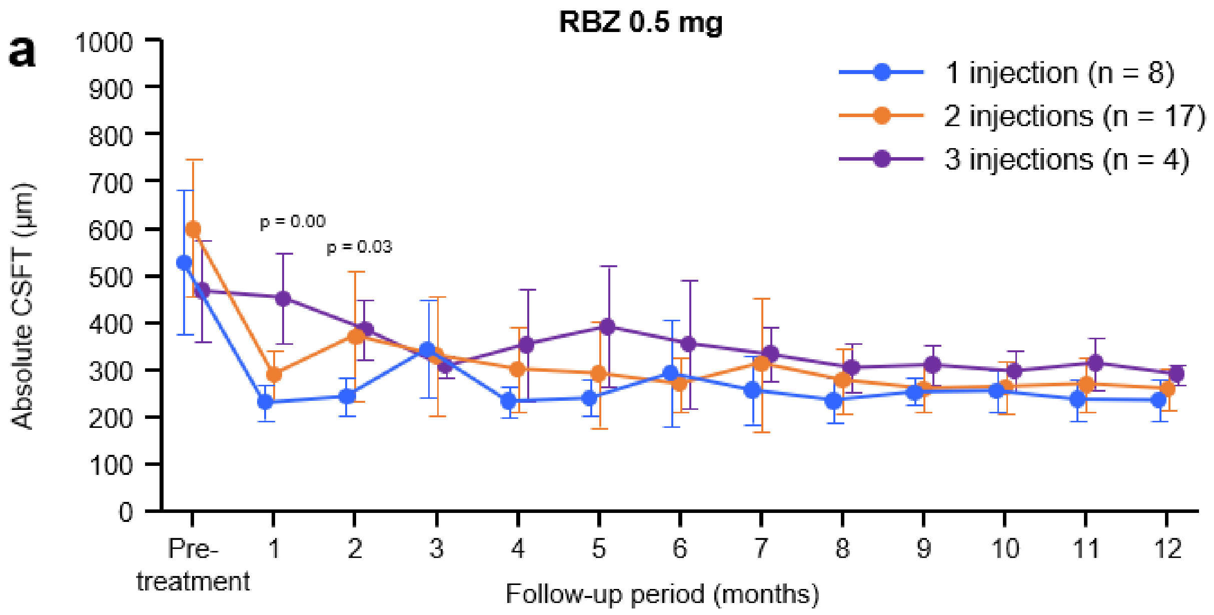

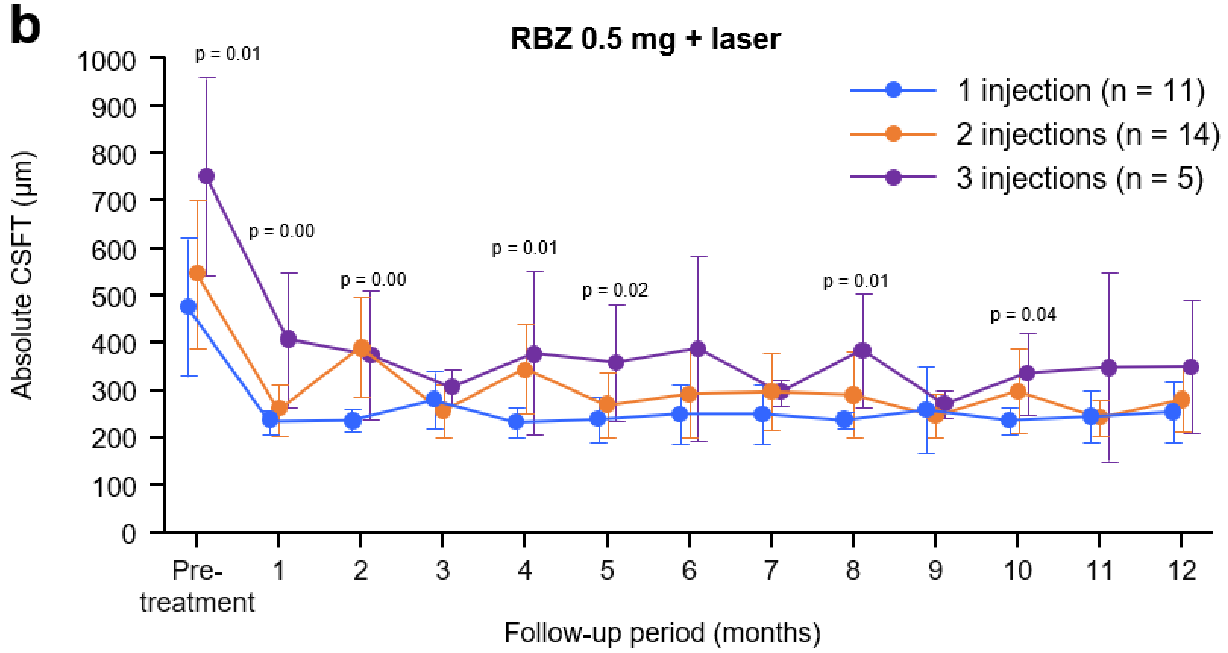

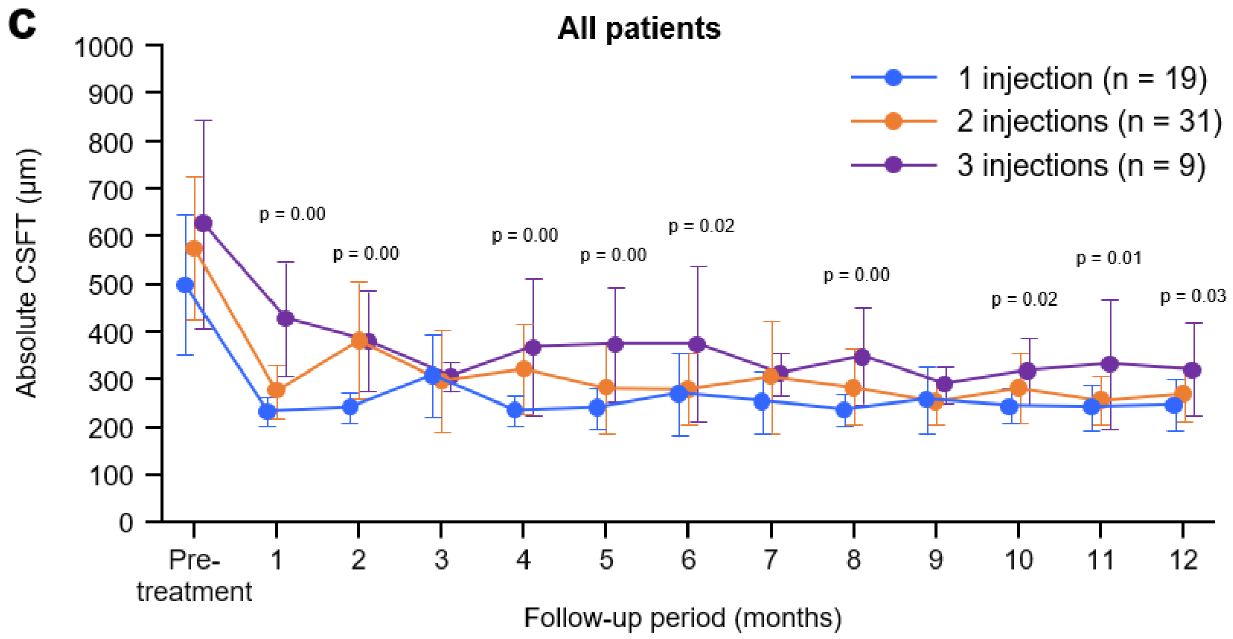

Supplementary Figure S1. Absolute CSFT values from baseline to Month 12. (**a**) RBZ 0.5 mg. (**b**) RBZ 0.5 mg + laser. (**c**) All patients. p values: 1 vs 2 vs 3 injections up to Month 2. Error bars represent 95% confidence intervals. *CSFT* central subfield foveal thickness, *RBZ* ranibizumab.
